# Response evaluation of hepatocellular carcinoma treated with stereotactic body radiation therapy: magnetic resonance imaging findings

**DOI:** 10.1007/s00261-023-03827-y

**Published:** 2023-03-20

**Authors:** Zhijun Mai, Qiuxia Yang, Jiahui Xu, Hui Xie, Xiaohua Ban, Guixiao Xu, Rong Zhang

**Affiliations:** Department of Radiology, Sun Yat-Sen University Cancer Center, No.651 Dongfeng Road East, Guangzhou, 510060 China

**Keywords:** Carcinoma, Hepatocellular, Magnetic resonance imaging, Stereotactic body radiation therapy, Contrast agent

## Abstract

**Purpose:**

To summarize the magnetic resonance imaging manifestations of hepatocellular carcinoma (HCC) with and without progression after stereotactic body radiation therapy (SBRT) and evaluate the treatment effect using the modified Liver Reporting and Data System (LI-RADS).

**Methods:**

Between January 2015 and December 2020, 102 patients with SBRT-treated HCC were included. Tumor size, signal intensity, and enhancement patterns at each follow-up period were analyzed. Three different patterns of enhancement: APHE and wash-out, non-enhancement, and delayed enhancement. For modified LI-RADS, delayed enhancement with no size increase were considered to be a “treatment-specific expected enhancement pattern” for LR-TR non-viable.

**Results:**

Patients were divided into two groups: without (*n* = 96) and with local progression (*n* = 6). Among patients without local progression, APHE and wash-out pattern demonstrated conversion to the delayed enhancement (71.9%) and non-enhancement (20.8%) patterns, with decreased signal intensity on T1WI(92.9%) and DWI(99%), increased signal intensity on T1WI (99%), and decreased size. The signal intensity and enhancement patterns stabilized after 6–9 months. Six cases with progression exhibited tumor growth, APHE and wash-out, and increased signal intensity on T2WI/DWI. Based on the modified LI-RADS criteria, 74% and 95% showed LR-TR-nonviable in 3 and 12 months post-SBRT, respectively.

**Conclusions:**

After SBRT, the signal intensity and enhancement patterns of HCCs showed a temporal evolution. Tumor growth, APHE and wash-out, and increased signal intensity on T2WI/DWI indicates tumor progression. Modified LI-RADS criteria showed good performance in evaluating nonviable lesions after SBRT.

**Supplementary Information:**

The online version contains supplementary material available at 10.1007/s00261-023-03827-y.

## Introduction

Stereotactic body radiation therapy (SBRT) is a promising and plausible locoregional treatment modality for patients with hepatocellular carcinoma (HCC) who are not eligible for curative treatment or other forms of locoregional treatment such as transarterial chemoembolization (TACE) and radiofrequency ablation [1–3]. SBRT enables the precise delivery of high-dose radiation to HCCs while sparing the adjacent hepatic parenchyma [4]. A growing number of studies have demonstrated that the use of SBRT provides good local control with a low risk of radiation-induced liver disease [5–8]. An understanding of the imaging appearance of HCC after SBRT and an accurate response assessment are critical for guiding clinical management. However, studies on imaging findings and treatment effect evaluation for HCCs post-SBRT remain limited.

Currently, the main algorithms used to evaluate tumor response after radiotherapy include the European Association for the Study of the Liver algorithm, Modified Response Evaluation Criteria in Solid Tumors (m-RECIST), and Liver Reporting and Data System treatment response algorithm (LI-RADS TRA). According to the m-RECIST, arterial phase hyperenhancement (APHE) on imaging is used as a main predictor for viable neoplasms [9, 10]. However, several studies have shown that the most successfully SBRT-treated HCCs demonstrate APHE for 3 months or more [11, 12]. Persistent APHE following SBRT is not necessarily suggestive of a viable tumor [11, 13]. Imaging features such as enhancement patterns evaluated via multiphase images, not just APHE assessed on arterial phase images, should be considered. For example, besides APHE, the LI-RADS TRA uses other imaging features, such as washout, depending on multi-phased images. In addition, an essential criterion for LR-TR nonviable tumors is the “treatment-specific expected enhancement pattern”. Our study focused on the magnetic resonance imaging (MRI) appearance of HCCs post-SBRT. Previous studies have evaluated the natural history of MRI features in SBRT-treated HCCs, although they were limited by small sample sizes [11, 14] and incomplete follow-up data in the early post-treatment period [12, 15]. Furthermore, the performance of current treatment response algorithms in HCC viability evaluation after SBRT should be assessed and compared.

Therefore, this study summarizes the MRI manifestations in patients with HCC with and without progression after SBRT, clarify the imaging appearance of HCC post-SBRT, and enable the early detection of tumor progression, thereby improving current imaging criteria for the response evaluation of HCCs post-SBRT.

## Materials and methods

### Patient selection

This study was approved by our institutional review board, and informed consent was not required in accordance with the requirements of a retrospective study.

Patients with HCC who underwent SBRT at our center between January 2015 and December 2020 were reviewed. We included patients: (1) with HCCs diagnosed by imaging appearance or biopsy; (2) who met the indications for SBRT and underwent complete SBRT procedures; (3) with HCC not treated using other locoregional treatments (e.g., TACE and radiofrequency ablation) within 3 months before SBRT; (4) who underwent MRI within 1 month before SBRT; and (5) who underwent MRI regularly after SBRT, with a follow-up duration ≥ 6 months. We excluded patients: (1) without baseline MRI data or in whom the interval from pre-SBRT MRI to SBRT initiation was > 1 month; (2) in whom the interval from SBRT completion to the first follow-up MRI was > 3 months or the follow-up duration was < 6 months; (3) who underwent a incomplete SBRT procedure; and 4) who had undergone transarterial radioembolization in the SBRT-treated segment, regardless of the timeframe. During the study period, 300 patients underwent SBRT.

**Fig. 1:**
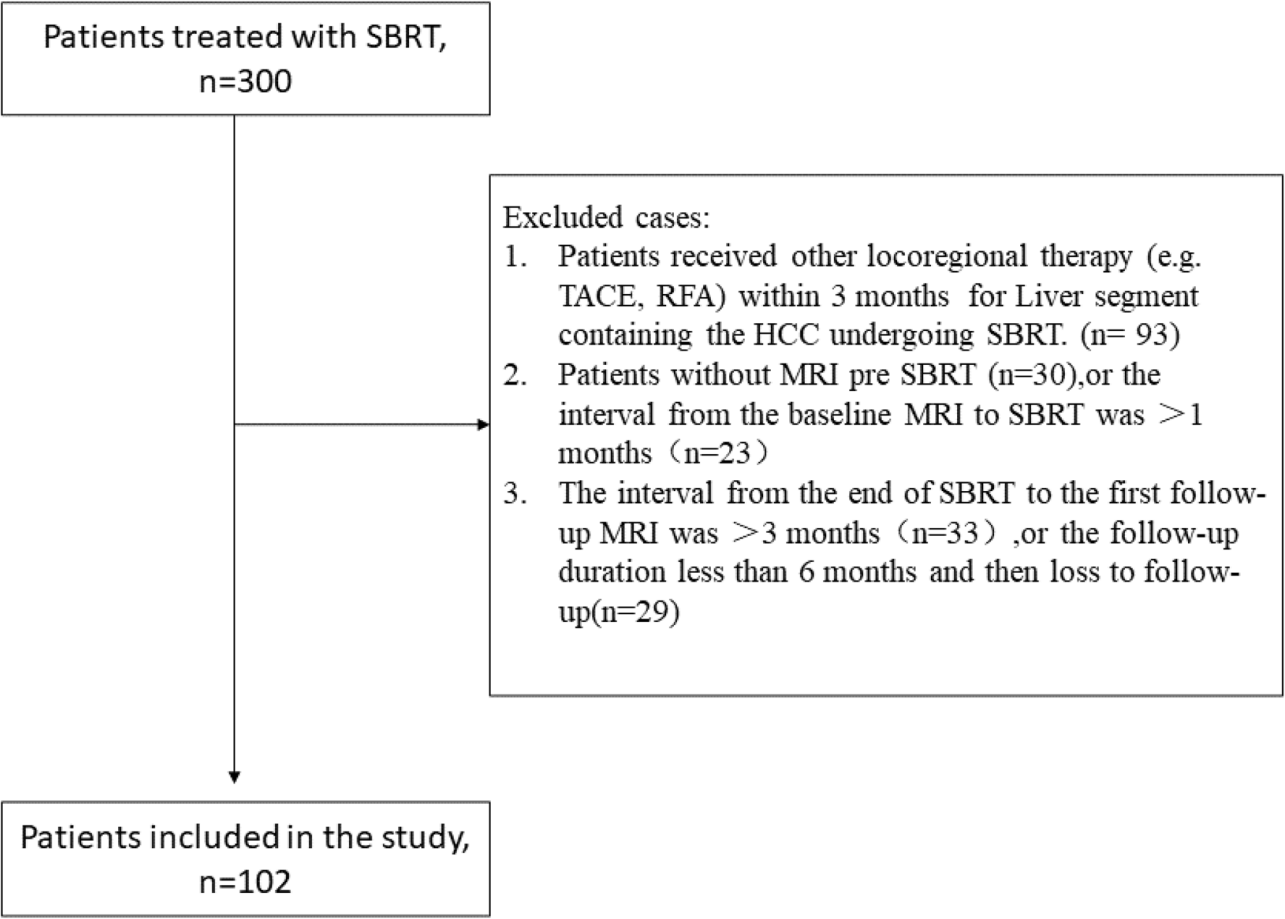
Patient selection process. *SBRT*, stereotactic body radiation therapy; *TACE* transarterial chemoembolization; *RFA* radiofrequency ablation; *MRI* magnetic resonance imaging; *HCC* hepatocellular carcinoma

A flow chart of patient selection is shown in Fig. 1. Ultimately, 102 patients with 102 SBRT-treated HCCs were included in the analysis. Each patient had one HCC treated with SBRT. Data regarding the patients’ age, sex, causes of cirrhosis, Child–Pugh class, previous treatment, baseline and follow-up alpha-fetoprotein (AFP) levels, and SBRT dose/fractionation were obtained from medical records.

### Imaging techniques

Details of MRI acquisition are provided in Supplementary Material 1.

MRI following SBRT was generally performed at 1, 3, 6, 9, and 12 months after the procedure for the first year and at intervals of 3–6 months after 1 year.

### SBRT techniques

Details of SBRT techniques are provided in Supplementary Material 2. The decision to treat HCC with SBRT was evaluated by a multidisciplinary liver tumor board.

### Imaging analysis

Imaging interpretation was performed on a picture archiving and communications system workstation (GE) by two radiologists (specialists in liver imaging with 3 years and 10 years of experience) independently, who were aware that the patients had HCC but were blinded to all other information, including clinical history and prognosis. In cases of disagreement between the two radiologists, a review was performed by a radiologist with over 30 years of experience in liver imaging until a consensus was reached.

In the pre- and post-treatment examinations, tumors were analyzed in terms of location; size (maximum diameter measured on the transverse plane); signal intensity on T1-weighted imaging (T1WI), T2-weighted imaging (T2WI), and diffusion-weighted imaging (DWI); and dynamic post-contrast imaging findings, including the presence of APHE and patterns of enhancement. Patterns of enhancement were categorized into three types based on enhancement characteristics on multi-phased imaging: APHE and washout, non-enhancement, and delayed enhancement. The APHE and washout pattern was defined as APHE and hypointensity on portal venous phase or delayed phase imaging. The non-enhancement pattern was defined as non-enhancement at each phase. On gadopentetate dimeglumine-enhanced MRI, the delayed enhancement pattern was defined as APHE with persistent delayed phase enhancement or arterial phase hypoenhancement with increasing delayed phase enhancement. On gadoxetic acid-enhanced MRI, the delayed enhancement pattern was defined as APHE with persistent venous phase enhancement or arterial phase hypoenhancement with increasing venous phase enhancement. T1WI signal intensity was categorized into three types: hypointensity, iso-hypointensity, and isointensity with respect to the surrounding untreated parenchyma. Iso-hypointensity refers to the signal intensity between hypointensity and isointensity. T2WI and DWI signal intensities were categorized into three types: hyperintensity, iso-hyperintensity, and isointensity. Iso-hyperintensity connotes the signal intensity between hyperintensity and isointensity. The imaging appearance of the surrounding post-treatment hepatic parenchyma was also evaluated. The abovementioned imaging findings were recorded and analyzed.

### Definition of tumor progression

Tumor progression was evaluated and defined for lesions meeting the following criteria: occurred during the post-SBRT follow-up period; demonstrated signal intensity and enhancement patterns (APHE and wash-out) corresponding to viable neoplasms; were enlarged, with a maximum diameter > 5 mm larger than the previously measured diameter; the AFP level was higher than that measured previously (for patients with a baseline AFP level elevation) or AFP levels exceeding normal values (for those with normal baseline AFP levels); or pathologically proven HCCs. New lesions at the SBRT site (apart from the original tumor site) and elsewhere within the liver were not classified as progressive tumors. The interval from the last SBRT session to tumor progression was recorded. The imaging features of the progressive tumors were analyzed.

The grouping criteria were as follows: tumors with progression post-SBRT were included in the group with local progression, while tumors without progression were included in the group without progression.

### Treatment response evaluation

Tumors were assessed using the m-RECIST [9] (complete response [CR], partial response [PR], progressive disease [PD], and stable disease [SD]), original LI-RADS TRA version 2018, and modified LI-RADS TRA [16] (viable [LR-TR viable], equivocal [LR-TR equivocal], and nonviable [LR-TR nonviable]) at each post-SBRT time point. The LI-RADS TRA version 2018 was used to categorize the treatment response of HCCs after SBRT as LR-TR equivocal. Therefore, lesions post-SBRT showing APHE and washout without increase in tumor size were categorized as LR-TR equivocal. More importantly, we integrated the definition of post-treatment imaging findings, based on the original LI-RADS TRA, into the modified LI-RADS TRA. Hence, lesions exhibiting delayed enhancement without increase in size were defined as having a “treatment-specific expected enhancement pattern,” and therefore were categorized as LR-TR nonviable in the modified LI-RADS TRA. Further details are shown in Supplementary Material 3.

### Data analysis

Continuous variables were reported as the mean ± standard deviation when variance analysis was used or as the median and range when the non-parametric Kruskal–Wallis test was used. Summary statistics were used to present the proportion of HCCs that showed different imaging findings in the pre- and post-SBRT examinations. The mean tumor size and AFP levels in the pre- and post-SBRT examinations were calculated. Linear weighted kappa (k) statistics were used to express interobserver agreement in imaging features (signal intensity and enhancement patterns) of SBRT-treated HCC. A two-by-two comparisons was made between the percentages of three enhancement patterns within group without progression. The original data were sampled one thousand times using the bootstrap method, and the statistical percentages were tested with t-test. Statistical analysis was performed using SPSS version 22.0 software (Chicago, IL, USA) and R software (version 4.0.4). All statistical tests were bilateral, and a *p*-value < 0.05 was considered significant.

## Results

### Patients

A total of 102 patients (85 men, 17 women; mean age, 53.8 ± 10.4 years [range, 28–79 years]) with 102 HCCs were included. The details of the study population are presented in Table 1. The mean tumor size was 22.2 ± 10.0 mm (median, 18.5 mm; range, 7–50 mm).

**Table 1:** Characteristics of the patients

Characteristics	group without progression (*N* = 96)	group with progression (*N* = 6)	*p*
Age, average (range), years	54.3 ± 10.4(28–79)	45.7 ± 5.4 (36–54)	0.05
*Sex, n (%)*			1
Male	80(83.3%)	5(83.3%)	
Female	16(16.7%)	1(16.7%)	
*Cause of cirrhosis, n (%)*			0.878
HBV	92(95.8%)	6 (100%)	
HCV	2(2.1%)	0	
Alcohol	2(2.1%)	0	
*Child–Pugh class, n (%)a*			NA
A	96(100%)	6 (100%)	
*Follow-up imaging, median (range), months*	15(6–48)	12 (6–52)	0.721
*Number of MRI per patient (median and IQR)*	6(3–13)	5(3–13)	0.948
*Previous treatment, n (%)*			0.109
TACE	1 (1.0%)	1 (16.7%)	
RFA	9 (9.4%)	1 (16.7%)	
RFA + TACE	3 (3.1%)	1 (16.7%)	
Segmentectomy	13 (13.5%)	1 (16.7%)	
Segmentectomy + RFA	7 (7.3%)	0	
Segmentectomy + TACE	6 (6.3%)	0	
Segmentectomy + TACE + RFA	10 (10.4%)	0	
None	47 (49.0%)	2 (33.3%)	
*Maximum tumor diameter, average(range), mm*	22.2 ± 10.2(7–50)	22.7 ± 7.4 (14–35)	0.526
*Number of tumors, n (%) *^b^			NA
Solitary	96(100%)	6 (100%)	
Multiple	0	0	
*Location*			0.718
Left lobe	27	2	
Right lobe	61	3	
Caudate lobe	8	1	
*AFP, median (range), IU/mL*^*c*^	877.2 ± 1146.5(40.9–4549.0)	NA	NA
*SBRT dose/fractionation, median (range)*			
Median SBRT physical dose	45 (36–57)	42 (39–54)	0.552
Median number of fractions^d^	3 (3–6)	3 (3–6)	NA

^ab^*p* values could not be calculated because the patients in two groups were all classified under Child–Pugh class A, with solitary tumors for which they underwent SBRT

^c^AFP levels were calculated in patients with baseline elevation. The *p* value could not be calculated for AFP levels because only one patient with tumor progression demonstrated baseline AFP level elevation

^d^*p* values could not be calculated because median number of fractions had no different in two groups

*SBRT* stereotactic body radiation therapy; *AFP* alpha-fetoprotein; *TACE* transarterial chemoembolization; *RFA* radiofrequency ablation; *MRI* magnetic resonance imaging; *IQR* interquartile range; *NA* not applicable

The median follow-up time was 15 months (range, 6–52 months). Seventy-eight and 70 patients had follow-up times > 6 months and ≥ 12 months, respectively. According to the diagnostic standard, the 102 lesions were divided into two groups: with (*n* = 6) and without (*n* = 96) local progression. The progression rate was 5.9% (6 cases).

### Tumor size in group without local progression

Ten of 96 (10.4%) HCCs increased in size in the initial months post-SBRT. These lesions either decreased in size (*n* = 9) or remained stable (*n* = 1) during the follow-up period.

**Fig. 2:**
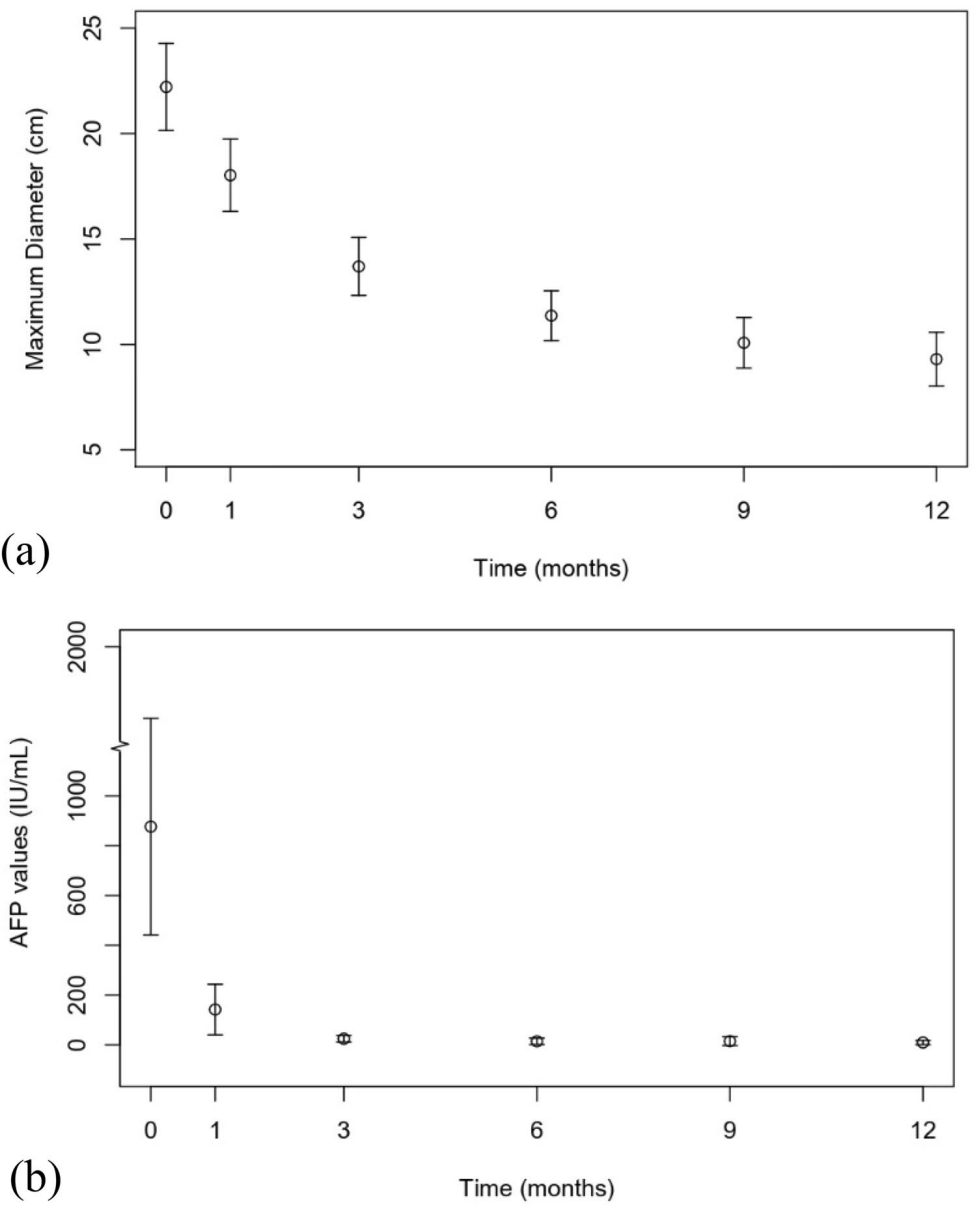
Change in maximum tumor diameter in the first 12 months for hepatocellular carcinoma (HCCs) undergoing stereotactic body radiation therapy (SBRT). **a** Change in maximum tumor diameter in the first 12 months for HCCs undergoing SBRT. **b** Change in alpha-fetoprotein (AFP) levels in the first 12 months for HCCs undergoing SBRT. Patients with a baseline elevation (AFP level > 25 IU/mL) were included. Dots represent means, while lines represent 95% confidence intervals

The most obvious HCC size reduction was observed within 3 months post-SBRT (Fig. 2a). During the 3-month follow-up MRI, the median HCC size reduction was 40.5% (mean, 36.0%), and the sizes of 67 of the 96 treated HCCs (69.8%) had decreased by ≥ 30%. By the end of the study period, the median proportion of maximum decrease was 60.0% (mean, 56.0; range, 9.0–86.0%). Seventy-two of 96 treated HCCs (75.0%) showed a size reduction ≥ 50%, while eighty-five of 96 showed a size reduction ≥ 30%. Moreover, 71 of 78 HCCs (91.1%) had stable sizes at 6 months post-SBRT, exhibiting a decrease in size ≤ 5 mm at the last follow-up compared with that measured at 6 months post-SBRT.

### Enhancement patterns in group without local progression

At baseline, all lesions exhibited APHE and washout. Within the first month post-SBRT, 59.4% (57/96) of the HCCs still exhibited APHE and washout. At 3 months, the proportion of HCCs exhibiting delayed enhancement (54.2% [52/96]) was higher than that of HCCs exhibiting APHE and washout (26.0% [25/96]). Meanwhile, *p* values derived from two-by-two comparisons between the three enhancement patterns were all < 0.05. From 6 months post-SBRT, the main enhancement pattern was converted to delayed enhancement, followed by non-enhancement.

**Fig. 3:**
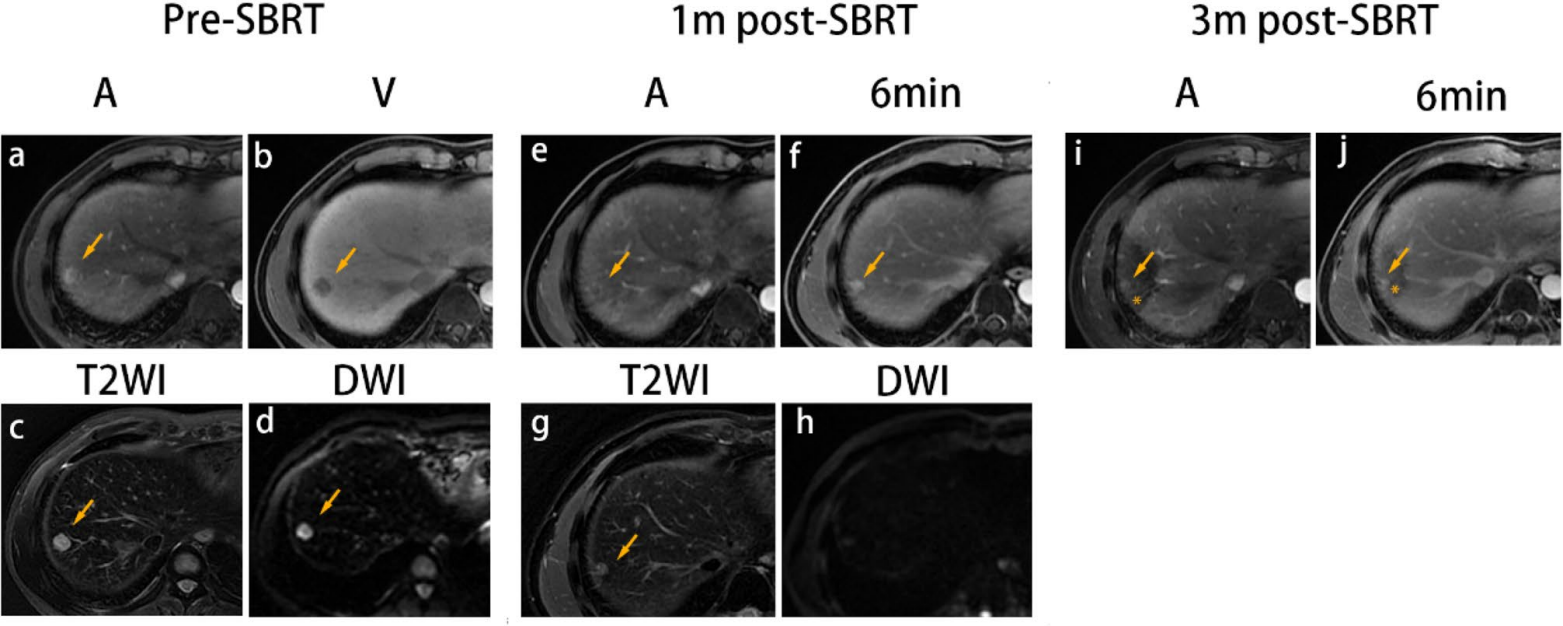
SBRT-treated HCC with conversion from APHE and washout before SBRT to delayed enhancement immediately after SBRT. A 44-year-old man with HBV-related cirrhosis and a 1.5-cm HCC in the seventh segment of the liver (arrow). At baseline, the lesion exhibited APHE and washout (**a**, **b**). Additionally, the lesion was hyperintense on T2WI (**c**) and DWI (d). At 1-month post-SBRT, the lesion demonstrated conversion to delayed enhancement (hypoenhancement on arterial phase imaging with increasing enhancement) (**e**, **f**) with a decrease in size, measuring 1.1 cm and a decrease in signal intensity on T2WI (**g**) and DWI (**h**). At 3 months post-SBRT, the lesion presented persistent delayed enhancement (**i**, **j**). Furthermore, at 3 months post-SBRT, there was wedge-like parenchymal arterial phase hypoenhancement and an increase in delayed phase images in the surrounding treated zone (asterisk). The lesion was categorized as CR and LR-TR nonviable based on m-RECIST and LI-RADS. *SBRT* stereotactic body radiation therapy; *A* arterial phase; *V* portal venous phase; *T2WI* T2-weighted imaging; *DWI* diffusion-weighted imaging; 6 min, delayed phase (6 min); *CR* complete response; *APHE* arterial phase hyperenhancement; *HCC* hepatocellular carcinoma; *m-RECIST* Modified Response Evaluation Criteria in Solid Tumors; *LI-RADS* Liver Reporting and Data System

**Fig. 4:**
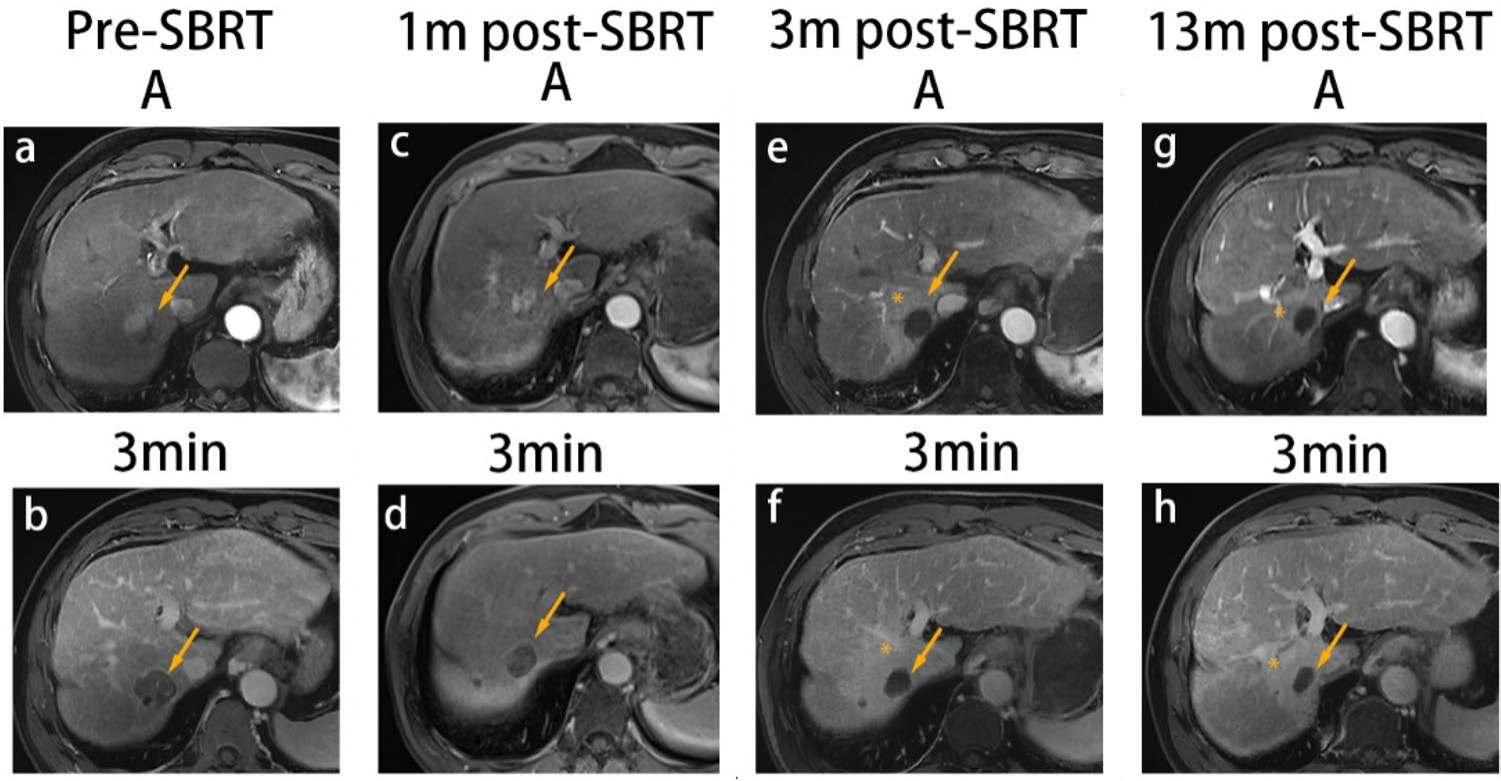
SBRT-treated HCC with conversion from APHE and washout pre-SBRT to non-enhancement immediately following SBRT. A 55-year-old man with HBV-related cirrhosis and a 3.0-cm cm HCC in the seventh segment of the liver (arrow). At baseline, the lesion exhibited APHE and washout (**a**, **b**). At 1-month post-SBRT, the lesion still exhibited APHE and washout with a decrease in size (**c**, **d**), measuring 2.5 cm. The lesion was categorized as PR and LR-TR equivocal based on m-RECIST and LI-RADS. At 3 months post-SBRT, the lesion had decreased in size, measuring 2.3 cm, with non-enhancement on multi-phased images (**e**, **f**), which persisted until the last follow-up MRI (13 months post-SBRT) (**g**, **h**). The lesion was categorized as CR and LR-TR nonviable based on m-RECIST and LI-RADS after 3 months. Additionally, at 3 months post-SBRT, there was a wedge-like parenchymal hyperenhancement with mild volume loss, which persisted until the last MRI (asterisk). *SBRT* stereotactic body radiation therapy; *A* arterial phase; 3 min, delayed phase (3 min); *PR* partial response; *CR* complete response; *APHE* arterial phase hyperenhancement; *HCC* hepatocellular carcinoma; *m-RECIST* Modified Response Evaluation Criteria in Solid Tumors; *LI-RADS* Liver Reporting and Data System; *MRI* magnetic resonance imaging

**Fig. 5:**
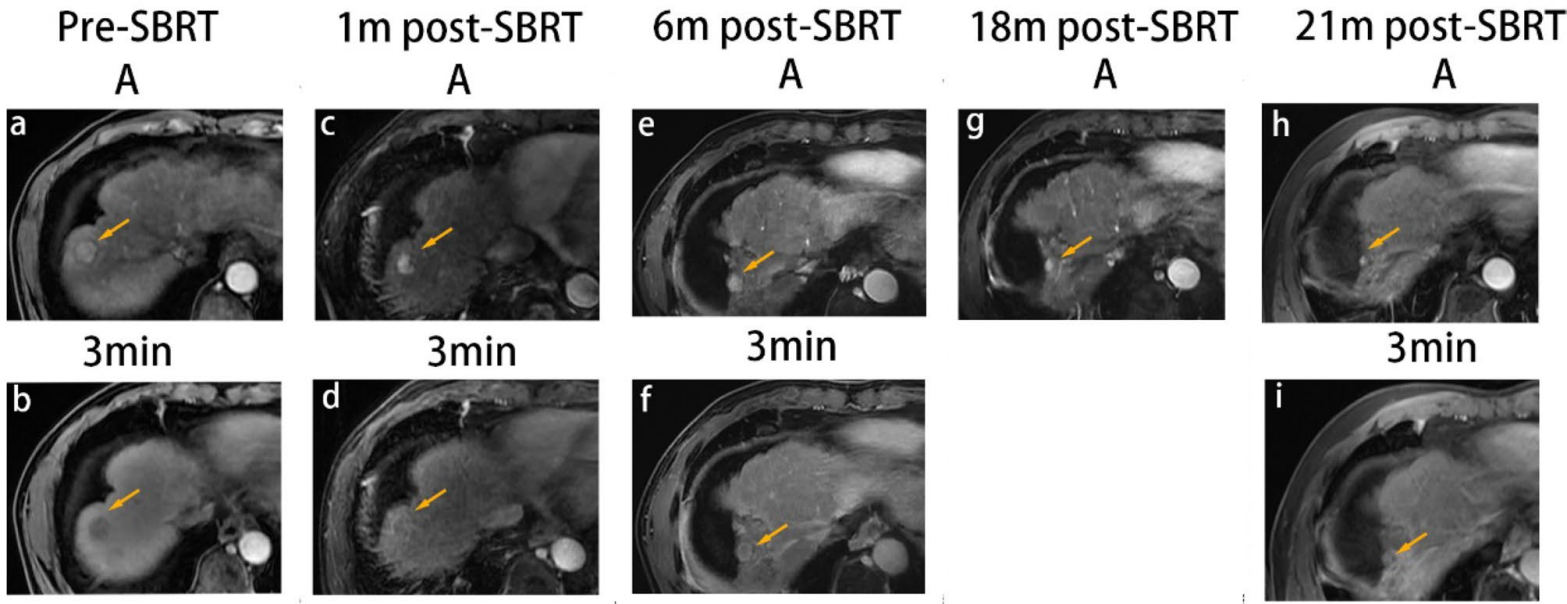
SBRT-treated HCC with persistent APHE and washout. A 66-year-old man with HBV-related cirrhosis and a 1.8-cm HCC in the eighth segment of the liver (arrow). At baseline, the lesion exhibited APHE and washout (**a**, **b**). At 1-month post-SBRT, the lesion exhibited APHE and washout (**c**, **d**) with a decrease in size, measuring 1.3 cm. The lesion showed persistent APHE and washout at 6 months (**e**, **f**) and 18 months post-SBRT (**g**). At the last follow-up MRI (21 months post-SBRT), the lesion showed persistent APHE and washout (**h**, **i**) with a size of 0.8 cm. Moreover, the surrounding parenchyma exhibited delayed phase enhancement with capsular retraction. The lesion was categorized as PR and LR-TR equivocal based on m-RECIST and LI-RADS after SBRT until the last follow up MRI. *SBRT* stereotactic body radiation therapy; *A* arterial phase; 3-min, delayed phase (3 min); *PR* partial response; *APHE* arterial phase hyperenhancement; *HCC* hepatocellular carcinoma; *m-RECIST* Modified Response Evaluation Criteria in Solid Tumors; *LI-RADS* Liver Reporting and Data System; *MRI* magnetic resonance imaging

Most (92.7% [89/96]) of the HCCs showed a conversion from the APHE and washout pattern to the other two enhancement patterns (Table 2), and the number of cases with pattern conversion within 3 months post-SBRT was the highest. At the end of the study, 71.9% (69/96) and 20.8% (20/96) of the HCCs demonstrated conversion to delayed enhancement (Fig. 3) and non-enhancement (Fig. 4) patterns, respectively, while seven cases demonstrated a persistence in the APHE and washout pattern until the time of the last follow-up (median, 9 months; range, 6–21 months) (Fig. 5). The median time for complete resolution of the APHE and washout pattern was 3 months (mean, 3.4 months; range, 1–21 months). In most (91.7% [88/96]) HCCs, the APHE and washout pattern persisted ≤ 6 months. The enhancement patterns became stable 9 months post-SBRT.

**Table 2:** Temporal evolution of MR imaging in tumors without progression

	Pre-SBRT *n* = 96	1 months *n* = 96	3 months *n* = 96	6 months *n* = 96	9 months *n* = 78	12 months *n* = 66	> 12 months *n* = 55
Enhancement patterns							
APHE and washout	96 (100)	57 (59)	25 (26)	11 (11)	4 (5)	3 (5)	1 (2)
Non-enhancement	0	10 (10)	19 (20)	20 (21)	14 (18)	12 (18)	12 (22)
Delayed enhancement	0	29 (30)	52 (54)	65 (68)	60 (77)	51 (77)	42 (76)
T1WI							
Hypointense	90 (94)	29 (30)	4 (4)	1 (1)	1 (1)	1 (2)	0 (0)
Iso-hypointense	6 (6)	67 (70)	70 (73)	70 (73)	57 (73)	49 (74)	43 (78)
Isointense	0	0	22 (23)	25 (26)	20 (26)	16 (24)	12 (22)
T2WI							
Hyperintense	93 (97)	44 (46)	9 (9)	3 (3)	1 (1)	0 (0)	0
Iso-hyperintense	3 (3)	46 (48)	62 (65)	49 (51)	34 (44)	27 (41)	20 (36)
Isointense	0	6 (6)	25 (26)	44 (46)	43 (55)	39 (59)	35 (64)
DWI							
Hyperintense	92 (96)	27 (28)	6 (6)	0	0	0	0
Iso-hyperintense	4 (4)	58 (60)	48 (50)	26 (27)	12 (15)	8 (12)	7 (13)
Isointense	0	11 (11)	42 (44)	70 (73)	66 (85)	58 (88)	48 (87)

Data are *N*(%). Percentile data may not sum to 100% due to rounding. Percentage data may not sum up to 100% due to approximations

*SBRT* stereotactic body radiation therapy; *APHE* arterial phase hyperenhancement; *MRI* magnetic resonance imaging; *T1WI*. T1-weighted imaging; *T2WI* T2-weighted imaging; *DWI* diffusion-weighted imaging

Details of hepatobiliary phase images after SBRT are provided in Supplementary Material 4.

### T1WI, T2WI, and DWI in group without local progression

Most HCCs (99.0% [95/96]) showed a progressive increase in signal intensity on T1WI. At the end of the study period, the signal intensities of 70 and 25 HCCs increased to iso-hypointensity and isointensity, respectively. The signal intensity on T1WI was stable after 6 months The median time for T1WI hypointensity was 1 month (mean, 1.9 months; range, 1–12 months). No tumor demonstrated decreased T1WI signal intensity.

Most (97.9% [94/96]) HCCs showed a progressive decrease in signal intensity on T2WI. Within the first month post-SBRT, the proportion of HCCs exhibiting iso-hyperintensity on T2WI (47.9% [46/96]) was slightly higher than that of HCCs exhibiting hyperintensity (45.8% [44/96]). Over time, the proportion of HCCs exhibiting T2WI isointensity progressively increased. From the 9-month follow-up MRI, T2WI isointensity became the main imaging finding, followed by iso-hyperintensity. The signal intensities of most HCCs (96.9% [93/96]) on T2WI were stable after 9 months post-SBRT and those of three HCCs were stable after 12 months. At the end of the study period, the signal intensities of 41 and 53 HCCs decreased to iso-hyperintensity and isointensity, respectively. The median time for T2WI hyperintensity was 1 month (mean, 2.4 months; range, 1–9 months). No tumor demonstrated increased T2WI signal intensity.

Most (99.0% [95/96]) HCCs showed a progressive decrease in signal intensity on DWI. Within the first month post-SBRT, most HCCs (60.4% [58/96]) showed iso-hyperintensity on DWI. Over time, the proportion of HCCs exhibiting DWI isointensity progressively increased. From the 6-month follow-up MRI, no lesion demonstrated DWI hyperintensity, and DWI isointensity became the main imaging finding, followed by iso-hyperintensity. The signal intensity on DWI was stable after 9 months. At the end of the study period, the signal intensity of 14 and 81 HCCs decreased to iso-hyperintensity and isointensity, respectively. The median time for DWI hyperintensity was 1 month (mean, 1.8 months; range, 1–6 months). No tumor demonstrated increased DWI signal intensity.

Details of the changes in signal intensity on T1WI, T2WI, and DWI are shown in Supplementary Material 5.

### Lesions with local progression

Of the six progressive tumors, two were diagnosed by pathology after surgical resection and four were diagnosed using imaging appearance and AFP levels. The median time for tumor progression was 6 months (range, 6–20 months).

**Fig. 6:**
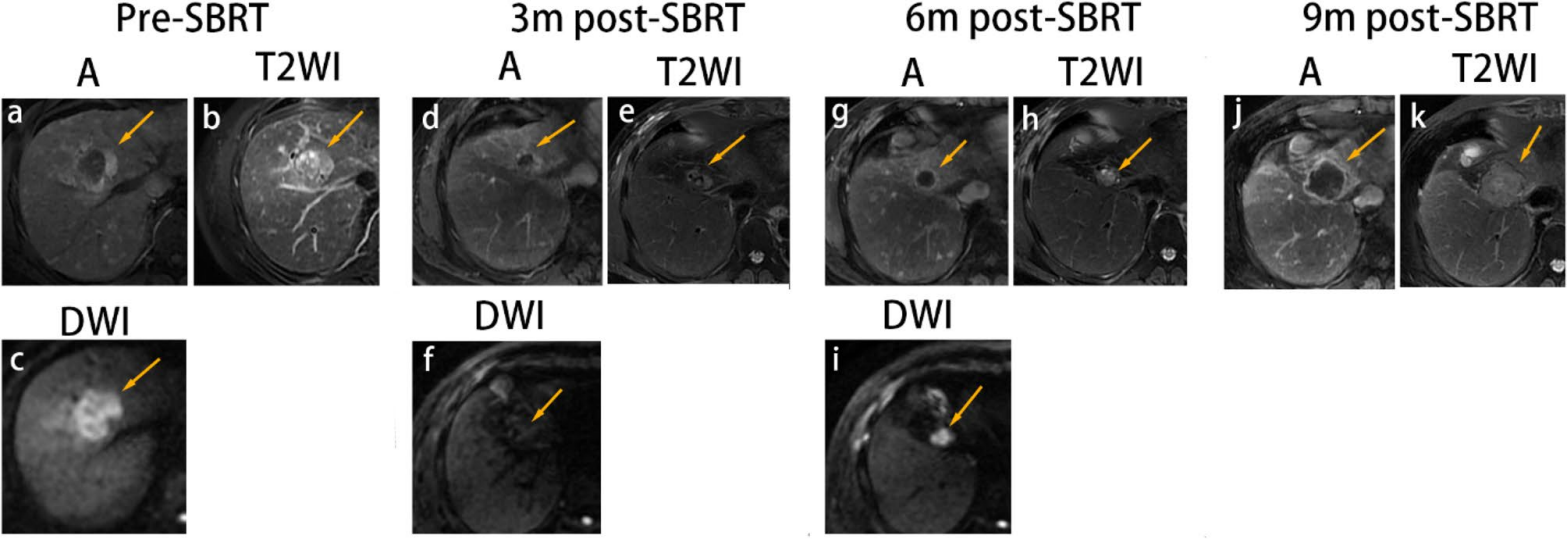
SBRT-treated HCC with persistent APHE and washout developed local progression. A 46-year-old man with HBV-related cirrhosis and a 3.5-cm cm HCC in the eighth segment of the liver (arrow). At baseline, the lesion exhibited APHE (**a**) with hyperintensity on T2WI (**b**) and DWI (**c**). At 3 months post-SBRT, the lesion showed APHE (**d**) with a decrease in size, measuring 1.7 cm, and iso-hyperintensity on T2WI (**e**) and DWI (**f**). At 6 months post-SBRT, the lesion increased in size, measuring 2.2 cm with annular enhancement (**g**) and hyperintensity on DWI (**i**) and T2WI (**h**). There was a decrease in wedge-like delayed phase parenchymal hyperenhancement with capsular retraction. The lesion was categorized as PD and LR-TR viable based on m-RECIST and LI-RADS. At the last follow-up MRI (9 months post-SBRT), the lesion continued to increase in size, measuring 4.0 cm (**j**, **k**). Eventually, the patient underwent surgical resection, and the lesion was confirmed pathologically. *SBRT* stereotactic body radiation therapy; *A* arterial phase; *T2WI* T2-weighted imaging; *DWI* diffusion-weighted imaging; *PD* progressive disease; *APHE* arterial phase hyperenhancement; *HCC* hepatocellular carcinoma; *m-RECIST* Modified Response Evaluation Criteria in Solid Tumors; *LI-RADS* Liver Reporting and Data System; *MRI*, magnetic resonance imaging

The specific imaging appearances of tumors with local progression are shown in Table 3. The imaging findings of these six progressive tumors before progression are shown in Supplementary Material 6. At the time of progression, these six tumors exhibited the following findings: tumor growth (median, 7 mm; range, 5–11 mm), increased T2WI and DWI signal intensities, unchanged T1WI signal intensity, and APHE and washout pattern (Fig. 6).

**Table 3:** Magnetic resonance imaging manifestations in recurrent tumors

Participant	Location	Size (mm)	AFP levels	Enhancement patterns	T2WI	DWI	Hepatobiliary phase
1							
Pre-SBRT	S2	19	858.4	APHE and wash-out	Hyperintensity	Hyperintensity	Hypointensity
1 month		13	68.27	APHE and wash-out	Iso-hyperintensity	Iso-hyperintensity	–
3 months		11	314.4	non-enhancement	Iso-hyperintensity	Iso-hyperintensity	–
6 months		11	448.1	non-enhancement	Iso-hyperintensity	Iso-hyperintensity	hypoinTensity
**9 months** *		**20**	**656.2**	**APHE and wash-out**	**Hyperintensity**	**Hyperintensity**	**Hypointensity**
12 months		23	2479	APHE and wash-out	Hyperintensity	Hyperintensity	Hypointensity
2							
Pre-SBRT	S1	18	6.27	APHE and wash-out	Hyperintensity	Hyperintensity	Hypointensity
1 month		16	12.23	APHE and wash-out	iso-Hyperintensity	Isointensity	–
3 months		9	19.61	APHE and wash-out	Isointensity	Isointensity	–
**6 months** *		**20**	**55.58**	**APHE and wash-out**	**Iso-hyperintensity**	**Iso-hyperintensity**	–
9 months		25	101.5	APHE and wash-out	Iso-hyperintensity	Iso-hyperintensity	–
12 months		28	421.2	APHE and wash-out	Hyperintensity	Hyperintensity	–
3							
Pre-SBRT	S8	24	4.01	APHE and wash-out	Hyperintensity	Hyperintensity	Hypointensity
1 month		20	6.43	APHE and wash-out	Iso-hyperintensity	Iso-hyperintensity	–
3 months		17	5.92	APHE and wash-out	Iso-hyperintensity	Isointensity	–
6 months		15	6.42	delayed enhancement	Isointensity	isointensity	–
9 months		14	5.49	delayed enhancement	Isointensity	Isointensity	–
18 months		13	4.42	delayed enhancement	Isointensity	Isointensity	–
20 months*		**22**	**4.04**	**APHE and wash-out**	**Isointensity**	**Isointensity**	–
28 months		27	4	APHE and wash-out	Iso-hyperintensity	Hyperintensity	Hypointensity
4							
Pre-SBRT	S7	14	1.14	APHE and wash-out	Hyperintensity	Hyperintensity	–
1 month		13	1.97	non-enhancement	Iso-hyperintensity	hyperintensity	–
3 months		12	1.86	non-enhancement	Iso-hyperintensity	Hyperintensity	Hypointensity
6 months*		**17**	**2.43**	**APHE and wash-out**	**Hyperintensity**	**Hyperintensity**	**Hypointensity**
5							
Pre-SBRT	S8	35	9.54	APHE and wash-out	Hyperintensity	Hyperintensity	–
1 month		30	13.27	APHE and wash-out	Hyperintensity	Iso-hyperintensity	–
3 months		17	9.49	APHE and wash-out	Iso-hyperintensity	Iso-hyperintensity	–
6 months*		**22**	**6.82**	**APHE and wash-out**	**Hyperintensity**	**Hyperintensity**	–
9 months		40	5.32	APHE and wash-out	Hyperintensity	Hyperintensity	–
6							
Pre-SBRT	S2	26	6.95	APHE and wash-out	Hyperintensity	Hyperintensity	Hypointensity
1 month		22	8.02	APHE and wash-out	Hyperintensity	Iso-hyperintensity	–
3 months		17	5.93	APHE and wash-out	Iso-hyperintensity	Iso-hyperintensity	–
6 months*		**22**	**3.64**	**APHE and wash-out**	**Iso-hyperintensity**	**Hyperintensity**	**Hypointensity**
9 months		24	3.3	APHE and wash-out	Hyperintensity	Hyperintensity	Hypointensity
12 months		26	2.39	APHE and wash-out	Hyperintensity	Hyperintensity	Hypointensity

*The bold parts of the table are indicative of the tumor progression time in the corresponding patient

*SBRT* Stereotactic body radiation therapy, *APHE* Arterial phase hyperenhancement, *AFP* alpha-fetoprotein, *T1WI* T1-weighted imaging, *T2WI* T2-weighted imaging, *DWI* diffusion-weighted imaging

The results showed excellent agreement between the two observers (κ = 0.85–0.95) (Supplementary Table 1).

### Treatment response evaluation

A total of 96 HCCs without progression were evaluated via the m-RECIST, LI-RADS TRA version 2018, and modified LI-RADS TRA, respectively (Table 4). Based on the m-RECIST, 10% (10/96) of the tumors met the CR criteria within the first month post-SBRT. Ten lesions met the PD criteria due to increase in size; 52% (50/96) and 64% (42/66) of the HCCs demonstrated CR at 6 and 12 months post-SBRT, respectively. The proportions of HCCs with PR + SD were 48% (46/96) and 36% (24/66) at 6 and 12 months, respectively.

**Table 4:** Response evaluation of patients after SBRT

	Group without progression				
	1 month *n* = 96	3 months *n* = 96	6 months *n* = 96	12 months *n* = 66	> 12 months n = 55
LI-RADS treatment response					
LR-TR nonviable	10 (10)	35 (46)	50 (52)	42 (64)	38 (69)
LR-TR equivocal	86 (90)	61 (64)	46 (48)	24 (36)	17 (31)
LR-TR viable	0	0	0	0	0
Modified LI-RADS treatment response					
LR-TR nonviable	39 (41)	71 (74)	85 (89)	63 (95)	54 (98)
LR-TR equivocal	57 (59)	25 (26)	11 (11)	3 (5)	1 (2)
LR-TR viable	0	0	0	0	0
m-RECIST					
Complete response	10 (10)	35 (36)	50 (52)	42 (64)	38 (69)
Partial response	27 (28)	37 (39)	34 (35)	20 (30)	15 (27)
Stable disease	49 (51)	22 (23)	12 (13)	4 (6)	2 (4)
Progressive disease	10 (10)	2 (2)	0	0	0

*SBRT* stereotactic body radiation therapy, *LI-RADS* Liver Reporting and Data System; *m-RECIST* Modified Response Evaluation Criteria in Solid Tumors

Besides, when using the original LI-RADS TRA version 2018 criteria for the 96 HCCs without progression, at 3 months, 46% (35/96) of the tumors met the criteria for LR-TR nonviable. The proportion of HCCs categorized as LR-TR nonviable reached up to 52% (50/96) and 64% (42/66) at 6 and 12 months post-SBRT, respectively.

Furthermore, based on the modified LI-RADS with new definition of LR-TR nonviable, At 3 months, 74% (71/96) of the tumors met the criteria for LR-TR nonviable. Over time, the proportion of HCCs categorized as LR-TR nonviable progressively increased, reaching up to 89% (85/96) and 95% (63/66) at 6 and 12 months post-SBRT, respectively.

### Surrounding parenchymal changes post-treatment

Within 1–3 months post-SBRT, 99% (101/102) of the tumors exhibited band-like or wedge-like changes. These post-treatment changes included hypointensity on T1WI and T2WI, mild APHE, and delayed phase hyperenhancement. Details are shown in Supplementary Material 7.

### AFP levels in patients without local progression

Seventy-two patients were treated for only one lesion and had no extrahepatic metastasis or intrahepatic progression through the course of follow-up. Among these patients, 30 had elevated AFP levels (> 25 IU/mL) at baseline with a mean value of 877.2 ± 1146.5 IU/mL (range, 40.9–4549.0 IU/mL). The temporal evolution of AFP levels is shown in Fig. 2b. The most obvious decline in AFP levels (median, 96.5%; mean, 93.9%; range, 55.6–99.8%) was observed within 3 months post-treatment. At the 15-month follow-up, the AFP levels of the 30 patients declined to normal values. The median time for normalization was 3 months (range, 1–15 months). The AFP levels rapidly decreased in patients with baseline AFP level elevation (Fig. 2b).

## Discussion

Imaging assessment is critical in patients with HCCs following different treatment modalities. After thermal ablation and TACE, the determination of viable tumors was mainly based on the presence of tumor vascularization. However, HCCs post-SBRT demonstrated different and complex imaging findings. We retrospectively analyzed the temporal evolution of MRI findings of SBRT-treated HCCs in 96 patients with and without tumor progression. Herein, we summarize the specific imaging features of various sequences in each follow-up period and the temporal evolution. Based on post-treatment imaging, we proposed the use of the modified LI-RADS TRA for image evaluation.

Among patients without local tumor progression, a few lesions showed a slight increase in size in the initial months post-SBRT, consistent with previous study findings [17, 18], possibly due to tumor hemorrhage or edema in the short-term post-treatment period [19].

In most patients without tumor progression, the signal intensity on T2WI/DWI decreased and that on T1WI increased after SBRT; these findings were similar to those from previous studies [12, 14, 15, 20]. A loss of cell membrane integrity and an increase in extracellular matrix distort the tissue structure, which eventually decreases DWI signal intensity [20, 21]. Moreover, in our study, the duration of DWI hyperintensity (mean, 1.8 months) was shorter than that of T2WI hyperintensity (mean, 2.4 months). Therefore, a decrease in DWI signal intensity may be a preferred early biomarker of HCC treatment efficacy [21, 22].

Combining enhancement characteristics on multi-phased imaging, three different patterns of enhancement for SBRT-treated HCCs were observed. The APHE and washout pattern remained the main enhancement pattern in the early post-SBRT period, consistent with previous study findings [11, 12, 14, 15]. Unlike other locoregional treatment modalities, SBRT does not induce immediate tumor necrosis or devascularization. Persistent APHE and washout may be caused by giant cell reactions, followed by progressive cell death and coagulation necrosis, resulting in a gradual decrease in the extent of enhancement [12, 19, 23, 24]. Thus, APHE and washout may not be suggestive of a residual tumor.

Previous studies on imaging findings have not confirmed that delayed enhancement is an important enhancement pattern for HCCs post-SBRT [11, 12, 14, 17]. Most of our study patients underwent delayed phase imaging at 6–7 min; therefore, we combined enhancement characteristics on arterial and delayed phase imaging to define two new enhancement patterns: non-enhancement and delayed enhancement patterns. SBRT-treated HCCs may be composed of varying degrees of coagulation necrosis and fibrosis, which exhibit delayed enhancement [19, 23]. Conversion to delayed enhancement may indicate successful treatment. Treatment efficacy may be more certain if these tumors demonstrate decreased T2WI/DWI signal intensity, increased T1WI signal intensity, and decreased size.

Among patients undergoing gadoxetic acid-enhanced MRI, viable lesions at baseline as well as lesions with and without progression exhibited hypointensity on hepatobiliary phase images. Therefore, hepatobiliary phase images may not help differentiate normal post-treatment changes from those of recurrent tumors. In contrast, some HCCs showed delayed enhancement on venous imaging without progression in the follow-up period and exhibited hypointensity on hepatobiliary phase images. This can cause a misinterpretation of successful treatment, leading to inappropriate re-treatment. Hence, the use of gadoxetic acid-enhanced MRI during follow-up should be further discussed, and gadoxetic acid-enhanced MRI images should be interpreted cautiously.

In our study, only a few lesions progressed (5.9%), similar to findings from previous studies, with tumor progression rates of 3.7% (1/27) [12], 6.25% (2/32) [16], and 11.1 (5/45) [19]. For progressive tumors, the conversion of enhancement patterns and signal intensity is accompanied by an increase in tumor size. Thus, for SBRT-treated HCCs, the following factors indicate the requirement of close monitoring for tumor progression: increase in tumor size accompanied with increased T2WI/DWI signal intensity and conversion of enhancement patterns from non-enhancement or delayed enhancement to APHE and washout. Hence, during the follow-up period after treatment, excessive clinical intervention is not recommended in the absence of tumor progression, regardless of the kind of enhancement patterns manifested.

The expected treatment-specific imaging features observed following SBRT differed from those after thermal ablation and TACE. Lesions effectively treated after thermal ablation demonstrate non-enhancement in the early post-treatment period [25]. However, we found that, at 12 months post-SBRT, nearly 40% of the lesions exhibited APHE due to giant cell reactions but not tumor viability [9]; based on the m-RECIST, these tumors were consistent with the criteria for PR or SD but not CR.

Thus, the m-RECIST may not be suitable for lesions showing persistent APHE. Compared with the m-RECIST that relied on APHE, the LI-RADS TRA was used to assess tumor viability based on multi-phased images. However, without definition of expected treatment-specific imaging features, when using original LI-RADS TRA, lesions exhibiting APHE were still categorized into LR-TR equivocal or viable. We proposed the use of the modified LI-RADS TRA after incorporating the definition of delayed enhancement without increase in tumor size as “treatment-specific expected enhancement pattern”. Based on the modified LI-RADS TRA, most tumors were categorized as LR-TR nonviable at the last follow-up, which was consistent with their clinical outcome of no progression after long-term follow-up. Therefore, we believe that the modified LI-RADS TRA may be preferable for HCCs post-SBRT. However, more clinical cases and prospective studies are needed to validate the modified LI-RADS TRA.

Our study has several limitations. First, our study was retrospective in nature. Regarding the three enhancement patterns we defined, a radiology-pathology correlation should be assessed. Second, some lesions underwent other locoregional treatments before SBRT. Although we excluded tumors that underwent locoregional treatments within 3 months before SBRT, it is unclear whether other locoregional treatments caused changes in tumor flow. Our study had the largest patient cohort to date, and we used MRI with various sequences. By comprehensively interpreting the imaging appearance of SBRT-treated HCCs and analyzing the temporal evolution, we obtained reliable results that help promote the development of novel imaging criteria for response evaluation in HCC post-SBRT.

In conclusion, after SBRT, the signal intensity and enhancement patterns of HCCs showed a temporal evolution. APHE and washout demonstrated progressive conversion to non-enhancement or delayed enhancement. The signal intensity and enhancement patterns stabilized after 6–9 months.. The use of the modified LI-RADS TRA, wherein we incorporated the definition of delayed enhancement without increase in tumor size as “treatment-specific expected enhancement pattern” showed good performance in evaluating nonviable lesions after SBRT.

## Supplementary Information

Below is the link to the electronic supplementary material.

Supplementary file1 (DOCX 28 KB)
