# Supplementary material for: Response evaluation of hepatocellular carcinoma treated with stereotactic body radiation therapy: magnetic resonance imaging findings
Source: Abdom Radiol (NY). 2023 Mar 20;48(6):1995–2007. doi: 10.1007/s00261-023-03827-y (PMC10167191; doi:10.1007/s00261-023-03827-y)
Supplement: Supplementary file 1 — Supplementary file1 (DOCX 28 KB) [file 261_2023_3827_MOESM1_ESM.docx]

**Response evaluation of hepatocellular carcinoma treated with stereotactic body radiation therapy： magnetic resonance imaging findings**

**Supplementary Information**

**Supplementary materials**

**1. Magnetic resonance imaging (MRI) and imaging parameters**

MRI was performed on a 1.5 Tesla (T) or 3.0 T system (Signa Horizon LX, HighSpeed; GE Healthcare, Milwaukee, Wisconsin; Ingenia CX, Philips, Netherlands) with the following sequences: fast spin echo T2-weighted in the axial plane and coronal plane, axial T1-weighted dual echo gradient recalled echo, axial T2-weighted with fat saturation, contrast-enhanced transverse T1-weighted, and axial diffusion-weighted imaging (DWI) with b-values of 0 and 800 s/mm^2^. The key parameters of sequences were shown in supplementary table 2. Contrast-enhanced transverse T1-weighted images with fat suppression were obtained (i.e., arterial [20–30 s], venous [60–90 s], and delayed phase [3 and 7 min]) after administering an intravenous injection of gadopentetate dimeglumine (Magnevist, Bayer Schering Pharma). To detect lesions and identify lesion viability, 42 patients at baseline and 19 patients during follow-up period underwent gadoxetic acid-enhanced MRI (Primovist, Bayer Schering Pharma). Hepatobiliary phase (15 and 20 min) images were obtained after the administration of an intravenous injection of gadoxetic acid (Primovist, Bayer Schering Pharma, 0.15ml/kg), followed by a 15-mL saline flush with an injection velocity of 1.0 mL/s. The three-dimensional contrast-enhanced T1-weighted imaging was obtained after intravenous injection of contrast material (gadopentetate dimeglumine, Bayer Schering Pharma AG, Berlin, Germany, 0.2 mmol/kg) followed by a 15-mL saline flush with an injection velocity of 2.0 ml/s.

**2. Stereotactic body radiation therapy (SBRT) techniques**

SBRT was indicated for all patients unsuitable for tumor resection or radiofrequency ablation, e.g., lesions near large vessels, and intrahepatic biliary and diaphragmatic surfaces, or lesions with associated tumor thrombus. All patients had Child-Pugh class A cirrhosis. Considering that tumors move 1–3 cm in size with respiration, patients were treated using a controlled breath-hold technique and abdominal compression to minimize movement, thereby sparing the surrounding normal hepatic parenchyma and achieving good local control rates with a low risk of radiation-induced liver disease. When deciding upon the treatment plan, by radiation oncologists, used pre-treatment CT images and MRI to delineate the gross tumor volume (GTV). The planning target volume was expanded by 5–10 mm from the GTV to account for uncertainties (e.g., changes in tumor size, shape, and site caused by respiratory movements) and ensure that the GTV receives the required dose of radiotherapy. Patients were treated with a 36–57 Gy dose in 3–6 fractions.

**3. Modified RECIST criteria and LI-RADS criteria**

Based on m-RECIST criteria, a complete response (CR) was defined as the disappearance of contrast enhancement in the tumor during the arterial phase. A partial response (PR) was defined as at least a 30% volume reduction of an enhanced area in the arterial phase, taking the baseline diameter of the tumor as a reference. A stable disease (SD) was defined as a tumor without any of these changes or an increase in volume. A progressive disease (PD) was defined as an increase of at least 20% in the diameter of a viable lesion.

Based on LI-RADS criteria, LR-TR nonviable was defined as no lesional enhancement or treatment-specific expected enhancement pattern. In our study, delayed enhancement was defined as a treatment-specific expected enhancement pattern, and therefore categorized as LR-TR nonviable. In addition, lesions showing non-enhancement were categorized as LR-TR nonviable. LR-TR equivocal was defined as enhancement atypical for treatment-specific expected enhancement pattern and not meeting the criteria for probably or definitely viable. According to current guidelines, lesions post-SBRT showing arterial phase hyperenhancement (APHE) and washout without increase in size were categorized as LR-TR equivocal. LR-TR viable was defined as APHE and washout appearance with increase in tumor size.

**4. Hepatobiliary phase images after SBRT**

At baseline, 42 patients underwent gadoxetic acid-enhanced MRI with, and their lesions exhibited hypointensity on hepatobiliary phase images (15 and 20 min). Furthermore, 19 patients underwent gadoxetic acid-enhanced MRI with throughout the course of follow-up; the total number of times was 40. The median follow-up time was 6 months (range,1–52 months). All lesions demonstrated hypointensity on hepatobiliary phase images post-SBRT. Of 19 HCCs, 11 showed delayed enhancement delayed phase images (3 min after contrast administration) and hypointensity on hepatobiliary phase images.

**5. Changes in signal intensity on T1WI, T2WI and DWI**

We observed the highest number of cases with T1-weighted signal intensity conversion within 3 months post-SBRT. Within the first month post-SBRT, most HCCs (69.8% [67/96]) showed iso-hypointensity on T1WI. After a 3-month follow-up MRI, T1-weighted iso-hypointensity became the main imaging finding, followed by isointensity. The proportion of HCCs exhibiting iso-hypointense and isointense lesions gradually increased within 6 months post-SBRT. Of the 25 HCCs demonstrating T1-weighted isointensity post-SBRT, six showed iso-hypointensity at baseline and one showed persistent hypointensity throughout the course of follow-up.

We observed the highest number of cases with T2WI signal intensity conversion within 3 months post-SBRT. At the last follow-up, out of 53 HCCs that demonstrated T2WI isointensity post-treatment, two showed iso-hyperintensity at baseline, of which one showed persistent hyperintensity and the other showed persistent iso-hyperintensity throughout the course of follow-up.

We observed the highest number of cases with DWI signal intensity conversion within 3 months post-SBRT. At the last follow-up, out of the 81 HCCs that demonstrated DWI isointensity post-SBRT, three showed iso-hyperintensity at baseline and one showed persistent iso-hyperintensity throughout the course of follow-up.

**6. Imaging findings before progression of group with local progression**

The following observations were noted in six lesions in group with progression before progression: the signal intensity on T2WI and DWI of four lesions decreased; the signal intensity on T1WI of five lesions increased, and one lesion demonstrated persistent T1-weighted hypointensity; the enhancement patterns of four lesions demonstrated conversions from APHE and washout to non-enhancement (n = 2) and delayed enhancement (n = 2) patterns; two lesions demonstrated persistent wash-in/wash-out.

**7. Details of surrounding parenchymal changes post-SBRT**

Simultaneously, 63.7% (65/102) and 24.5% (25/102) of the tumors showed biliary dilation, and capsular retraction at the SBRT treatment site, reapectively. At the 6-month follow-up MRI, 83.1% (84/101) of the tumors showed a reduction in the post-SBRT change zone compared to that during the last follow-up period. During the same period, 72.5% (74/102), 52.9% (54/102), and 47.1% (48/102) of the tumors revealed biliary dilation, capsular retraction, and narrowed portal/hepatic veins, reapectively. In the 40 hepatobiliary phase images obtained during the follow-up period, the signal intensity of the surrounding hepatic parenchyma was similar to that of the SBRT-treated HCCs.

| **Supplementary Table 1. Interobservers agreement** | | | | | |  |  |
| --- | --- | --- | --- | --- | --- | --- | --- |
|  | Baseline | 1month | 3months | 6months | 9months | 12months | ＞12months |
| Enhancement patterns | 0.85(0.57,1.00) | 0.91 (0.84,0.98) | 0.94 (0.88,1.00) | 0.93 (0.85,1.00) | 0.89 (0.78.1.00) | 0.9 (0.79,1.00) | 0.87 (0.73,1.00) |
| T1WI | 0.85 (0.64,1.00) | 0.93 (0.84,1.00) | 0.91 (0.87,0.96) | 0.9 (0.81,0.98) | 0.85 (0.73,0.98) | 0.86 (0.74,0.99) | 0.86 (0.70,1.00) |
| T2WI | 0.85 (0.60,1.00) | 0.93 (0.86,1.00) | 0.89 (0.79,0.97) | 0.91 (0.94,0.99) | 0.89 (0.80.0.98) | 0.92 (0.82,1.00) | 0.91 (0.80,1.00) |
| DWI | 0.89 (0.66,1.00) | 0.89 (0.81,0.98) | 0.89 (0.81,0.98) | 0.91 (0.83,1.00) | 0.9 (0.79,1.00) | 0.87 (0.72,1.00) | 0.89 (0.74,1.00) |

| Technique | axial T2-weighted | T2-weighted in the coronal plane | axial T1-weighted | axial T2-weighted with fat saturation | contrast-enhanced transverse T1-weighted | DWI with b-values of 0 and 800 s/mm2 |
| --- | --- | --- | --- | --- | --- | --- |
| Signa Horizon LX, HighSpeed; GE Healthcare (1.5T) | | | | | | |
| repetition time (ms) | 2200 | 536 | 6.93 | 2390 | 4.63 | 5600 |
| echo time (ms) | 66 | 91 | 2.39 | 69 | 2.21 | 49 |
| matrix | 384×384 | 320×256 | 320×240 | 320×272 | 320×272 | 128×100 |
| field of view (mm^2^) | 360×260 | 360×260 | 360×260 | 360×260 | 360×260 | 360×260 |
| section thickness (mm) | 5 | 5 | 3 | 5 | 3 | 5 |
| intersection gap (mm) | 1 | 1 | 0.6 | 1 | 0.6 | 1 |
| flip angle | 140 | 150 | 10 | 130 | 10 | None |
| parallel imaging factor | 2 | 2 | 2 | 2 | 2 | 2 |
| number of excitations | 1 | 1 | 1 | 2 | 1 | b=50,1; b=800,8 |
| Ingenia CX, Philips, Netherlands (3.0T) | | | | | | |
| repetition time (ms) | 1800 | 950 | 3.7 | 1850 | 3.7 | 1083 |
| echo time (ms) | 92 | 80 | 1.31 | 87 | 1.31 | 64 |
| matrix | 316×316 | 332×256 | 320×208 | 280×280 | 320×206 | 128×126 |
| field of view (mm^2^) | 380×380 | 400×400 | 400×340 | 380×380 | 400×340 | 380×380 |
| section thickness (mm) | 5 | 6 | 4 | 5 | 4 | 5 |
| intersection gap (mm) | 1 | 1 | -2 | 1 | -2 | 1 |
| flip angle | 90 | 90 | 16 | 90 | 15 | 90 |
| parallel imaging factor | 2 | 3.5 | P=2; S=1.6 | 2 | None | 2 |
| number of excitations | 1 | 1 | 1 | 1 | 1 | b=0,1; b=800,6 |

**Supplementary Table 2. Key parameters of sequences in 1.5 and 3.0 MRI**
